# Writing with a broken heart: Literature and the many faces of grief

**DOI:** 10.1017/S1478951526102569

**Published:** 2026-06-09

**Authors:** Eduardo Antuña-Zarzuelo, Victoria Lobo-Antuña, Marta Lobo-Antuña

**Affiliations:** 1 Legal Advisory Service of the Court of Accounts of Spain, Tribunal de Cuentas, Madrid, Spain; 2 Infectious Diseases Unit, Consorcio Hospital General Universitario de Valencia, Valencia, Spain; 3 Palliative Care Unit, Hospital Universitario Fundación Jiménez Díaz, Madrid, Spain

Human relationships are difficult. Beginnings should be easy, yet we often complicate them, and a single misstep can ruin everything. Maintaining relationships is even harder. And the end – inevitable as it is – is always the most painful.

This essay does not aim to analyze human relationships themselves, but rather the attitudes we adopt when facing their end: the loss of those we love. Literature offers countless examples of writing born from grief. Authors have long attempted to give language to loss, and in doing so they reveal different ways in which human beings confront absence.

In the following reflections, 4 writers – Cesare Pavese, Miguel Hernández, Julian Barnes, and Joan Didion – offer distinct responses to grief. Each writes from a broken heart, yet each reveals a different path through loss.

## Cesare Pavese: loss as resentment

Cesare Pavese’s diaries, published as *The Business of Living*, remain an essential reference for anyone interested in literary creation. They are filled with reflections on technique, translation, and artistic ambition. Yet within them lies something more raw: the record of a wounded heart.

In 1936, Pavese returned to Turin after being confined by Mussolini’s regime for antifascist activities. Upon his return, he learned that the woman he loved had married someone else. His friend reportedly told him simply: “Do not think about her anymore. She married yesterday morning.”

From that moment onward, Pavese’s diary entries darkened. His reflections became increasingly dominated by bitterness toward love and women. The personal wound evolved into a broader resentment that shaped his view of relationships.

At times the despair borders on self-destruction. On April 10, 1936, he writes: “Have I ever done anything in my life that was not foolish?” Soon afterward he confesses a terrifying thought: suicide as an ever-present possibility.

Over time, however, resentment itself becomes a form of survival. Hatred, harsh as it may appear, serves as a psychological armor protecting him from deeper collapse. Eventually the intensity subsides. By January 1, 1939, he writes that the year has been one of reflection and liberation. The wound has not disappeared, but it has changed shape.

Pavese’s response to loss reminds us that grief does not always appear in noble forms. Sometimes it manifests as anger, bitterness, or resentment – emotions that may seem troubling but that nevertheless represent an attempt to endure pain.

## Miguel Hernández: loss as tears

Miguel Hernández represents almost the opposite response to grief. If Pavese turned toward resentment, Hernández turned toward tears.

Hernández’s life was marked by hardship. After finally finding love with Josefina and the birth of his son, the Spanish Civil War shattered his life. Imprisoned by the Franco regime, he wrote some of the most moving poetry in Spanish literature.

Two poems written earlier, however, capture grief in its most immediate form: the elegies dedicated to Ramón Sijé and Federico García Lorca.

In the elegy for Sijé, Hernández writes one of the most powerful lines in Spanish poetry:

So much pain gathers in my side that even my breath hurts.

The poem becomes a cry against death itself:

I do not forgive death in love, I do not forgive inattentive life, I do not forgive earth nor nothingness.

The rejection of death reappears in the elegy for Lorca, where the poet denounces the sudden brutality with which death strikes.

Yet Hernández’s grief also contains a remarkable vitality. Toward the end of the elegy for Sijé he imagines digging through the earth to recover his friend and bring him back to life. The poem ends not in resignation but in an intimate call to continue their conversation: “*We still have many things to talk about, my friend.*”

In Hernández’s work, grief becomes an overwhelming emotional force expressed through language. Tears are not weakness; they are testimony to love.

## Julian Barnes: loss as the persistence of grief

In *Levels of Life*, Julian Barnes reflects on the death of his wife, Pat Kavanagh. The book is not merely about grief but about the way grief reshapes time, memory, and identity.

Barnes and his wife had been together for 30 years. After her death, he confronts a question that many bereaved people face: must life continue in the same way? Must one “move on”?

Barnes resists this expectation. Grief, he suggests, is not something to be overcome quickly. It is a transformation of the inner landscape.

“The pain,” he writes, “is directly proportional to the value of what has been lost.”

Time itself changes under grief’s influence. Days lose their structure. The world outside appears strangely irrelevant. Barnes confesses that after his wife’s death he struggled to care about anything beyond the fact that she was gone.

Yet within this persistent grief lies a paradox: pain becomes a form of remembrance. To continue loving the person who has died is also to continue feeling the pain of their absence.

In this sense, grief becomes a proof of love.

## Joan Didion: loss as narrative

Few writers have explored grief with the clarity and honesty of Joan Didion. In *The Year of Magical Thinking* and *Blue Nights*, she recounts the devastating loss of her husband and, shortly afterward, her daughter.

Didion describes the sudden death of her husband in their home one evening in 2003, while their daughter lay critically ill in the hospital. Two years later, the daughter also died.

The books that followed are attempts to understand what happened. Yet Didion recognizes that language itself struggles to capture the experience of loss.

“I write,” she explains, “to understand what happened.”

For Didion, storytelling becomes a way to confront grief. Memory is dissected, revisited, examined from every angle. The act of narrating becomes both analysis and survival.

She describes grief as a kind of altered state in which rational thought breaks down. Even simple routines become difficult. Identity itself changes: she struggles to think of herself as a widow.

Yet writing allows her to preserve the presence of those she has lost. Memory resists disappearance. Through narrative, the past continues to speak.

## Literature and the human experience of grief

These 4 writers show that grief has no single form. It may appear as resentment, as tears, as a prolonged attachment to pain, or as an effort to preserve memory through narrative.

What unites them is the impulse to write.

Writing does not erase loss. It cannot restore what has disappeared. But it allows the bereaved to shape their experience into meaning, to transform private pain into something that can be shared and understood.

In this sense, literature becomes not only an artistic act but also a profoundly human one. When writers speak from a broken heart, they illuminate the universal experience of loss – and remind us that grief, however painful, is inseparable from love.

